# Accuracy of the GPT-5 Mini in Predicting Six-Week Postoperative Knee Flexion Following Total Knee Replacement: A Retrospective Cohort Study

**DOI:** 10.7759/cureus.102196

**Published:** 2026-01-24

**Authors:** Matthew Earnshaw, Hassan Shadad, Abed Alfattah Mahmoud Alnsour, Damien Mony, Afolabi Benjamin Olapade-Ayomidele, Usama Yaseen, Ihab Boutros

**Affiliations:** 1 Trauma and Orthopaedics, University of Manchester, Manchester, GBR; 2 Trauma and Orthopaedics, Salford Royal NHS Foundation Trust, Salford, GBR; 3 Urology, Royal Hallamshire Hospital, Sheffield, GBR

**Keywords:** artifical intelligence, chatgpt-5, knee function, post-operative analysis, retrospective research, total knee replacement (tkr)

## Abstract

Background and objective: Artificial intelligence (AI) models such as ChatGPT are increasingly explored for clinical prediction, yet their accuracy in forecasting early functional outcomes after total knee replacement (TKR) remains unclear. This study aims to evaluate the accuracy of the ChatGPT platform via the GPT-5 mini model (OpenAI, San Francisco, CA, USA) in predicting six-week postoperative knee flexion following TKR and assess whether patient factors influence prediction error.

Methods: This retrospective cohort study included 160 patients who underwent TKR at a UK tertiary center. Age, sex, BMI, diabetic status, smoking status, American Society of Anaesthesiologists (ASA) grade, and six-week postoperative knee flexion were extracted from electronic records. The GPT-5 mini generated predicted flexion values using a standardized prompt. Predicted and actual flexion were compared using the Wilcoxon signed-rank test. Agreement was evaluated using Bland-Altman analysis. Subgroup analyses assessed age, diabetes, smoking, ASA grade, and BMI.

Results: Median actual flexion was 95°, while the median GPT-5 mini predicted flexion was 103° (p < 0.0001). Median absolute error was 10°. Significant overestimation occurred across most age groups, diabetic and non-diabetic patients, smokers and non-smokers, and all ASA grades. Absolute error differed significantly by ASA grade (I: 17°, II: 9°, III: 6°, p < 0.001). The BMI showed no association with prediction error.

Conclusion: The GPT-5 mini overestimated six-week postoperative flexion, with the greatest inaccuracies occurring in younger, healthier patients and smaller errors observed in those with higher comorbidity burden. Thus, GPT-5-mini is not reliable and should not be used clinically without rigorous validation on institution-specific datasets.

## Introduction

Total knee replacement (TKR) is an effective treatment for advanced knee osteoarthritis and is performed with increasing frequency worldwide [1]. Despite high overall success rates, postoperative recovery varies substantially, and early functional outcomes strongly influence patient satisfaction [2]. Age, comorbidities, and lifestyle factors are known to affect postoperative pain, mobility, and recovery trajectories [3-5].

Artificial intelligence (AI) and machine learning have gained attention for their potential to improve outcome prediction by identifying complex patterns within clinical data [6,7]. Prior studies have demonstrated the feasibility of using AI to predict postoperative range of motion and patient satisfaction [6]. However, the use of large language models (LLMs), such as ChatGPT, for generating numerical predictions of early postoperative functional measures remains largely untested. This study evaluated the accuracy of GPT-5 mini (ChatGPT; OpenAI, San Francisco, CA, USA) in predicting six-week postoperative knee flexion using routine patient data and examined whether patient factors such as age, diabetes, smoking status, BMI, and American Society of Anaesthesiologists (ASA) grade affect prediction error.

## Materials and methods

This retrospective cohort study was conducted at Salford Royal Hospital, a tertiary care center in the United Kingdom. A total of 160 patients who underwent primary TKR between January 4, 2024, and February 25, 2025, were included consecutively. Patients were included if six-week postoperative knee flexion measurements were available and were excluded if there were incomplete records or TKR performed for traumatic indications. No patient met the exclusion criteria, and therefore all 160 patients were included in the study.

Six-week postoperative knee flexion values were measured visually by an experienced surgeon during outpatient follow-up by asking the patient to flex and extend at the knee. No formal inter- or intra-observer reliability assessment was performed. Electronic health records were reviewed to obtain age, sex, BMI, smoking status, diabetic status, and the American Society of Anaesthesiologists (ASA) grade [8].

Prediction procedure by GPT-5 mini

The GPT-5 Mini (ChatGPT; OpenAI, San Francisco, CA, USA), released in January 2025, was accessed via the web-based interface. Default model settings were used, and no external datasets were provided to GPT-5 mini. For each patient, the following standardised prompt was used to generate a singular flexion value for each patient: “A [age]-year-old [sex] patient (BMI [value], ASA grade [I-III], [smoker/non-smoker], [diabetic/non-diabetic]) underwent total knee replacement. What is the expected knee flexion at 6 weeks postoperatively?” Each patient was entered into a new GPT-5 mini session. The first numerical flexion value that was output by GPT-5 mini was manually collected and treated as the predicted flexion (°). Predictions were collected by a singular assessor who was not blinded to the actual flexion measurement undertaken in the clinic.

Data protection and information governance

To comply with general data protection regulations, no identifiable patient information, such as patient name, NHS number, date of surgery, free-text clinical records, or other explicit patient identifiers, was entered into GPT-5 mini. Only anonymized, categorical, or numerical variables were used. Formal ethical approval was not required, as the study involved analysis of anonymized, routinely collected clinical data that was confirmed by the clinical governance lead at Salford Royal Hospital. 

Statistical analysis

All statistical analyses were performed using SPSS Statistics version 29.0.1.0 (IBM Corp., Armonk, NY, USA) and GraphPad Prism version 10.6.0 (GraphPad Software, San Diego, CA, USA). Data distribution was assessed using the Shapiro-Wilk test. As both actual and predicted flexion values were non-normally distributed, non-parametric tests were used.

Actual and predicted flexion values were compared using the Wilcoxon signed-rank test. A Bland-Altman analysis [9] was used to assess bias and limits of agreement. Absolute error was defined as follows: |predicted postoperative flexion − actual postoperative flexion|. Subgroup analyses were prespecified based on known predictors of TKR postoperative recovery. Age group, smoking, and diabetic status subgroup analyses of actual flexion vs. AI-generated flexion were compared using the Wilcoxon signed-rank test. The ASA grade (≥3 groups) of absolute error was compared using the Kruskal-Wallis test. The relationship between BMI and absolute error was tested using the Spearman rank correlation. No formal correction for multiple testing was applied due to the exploratory nature of the study. A p-value of less than 0.05 was considered statistically significant. 

## Results

Patient characteristics

Data from 160 patients were collected. These patient characteristics are listed in Table 1. 

**Table 1:** Patient demographics ASA grade: American Society of Anaesthesiologists grade [8]

Parameter	Values
Age, median (range)	70.5 (41-87)
Sex, n (%)	Female: 96 (60.0%); male: 64 (40.0%)
BMI, mean (range)	31.58 (16.3-43.8)
Smoking status, n (%)	Non-smoker: 107 (67%); smoker: 53 (33%)
Diabetic status, n (%)	Non-diabetic: 139 (87%); diabetic: 21 (13%)
ASA grade, n (%)	Grade I: 37 (23.1%); grade II: 88 (55.0%); grade III: 35 (21.9%)

Overall AI prediction performance

**Figure 1:**
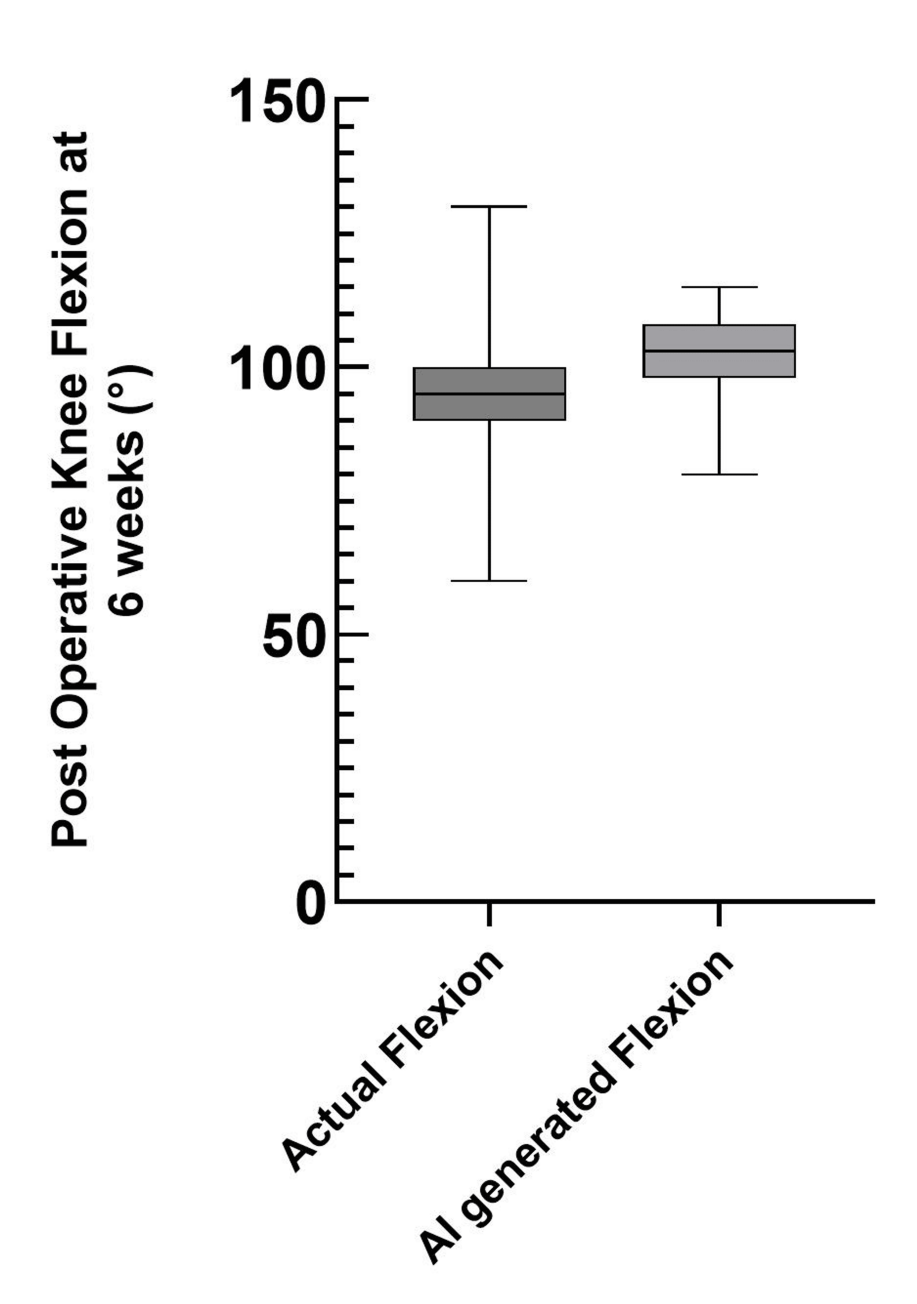
Comparison of actual and AI-predicted postoperative knee flexion at six weeks A box plot showing the median actual postoperative knee flexion (left) compared to the median GPT-5 mini-predicted postoperative knee flexion (right).

There was a significant difference between actual knee flexion and GPT-5 mini-predicted knee flexion (actual median 95° (95% CI 90-100) vs. predicted median 103° (95% CI 101-104); Wilcoxon p < 0.0001, |r| = 0.52) (Figure 1). Median absolute error was 10°. 

**Figure 2:**
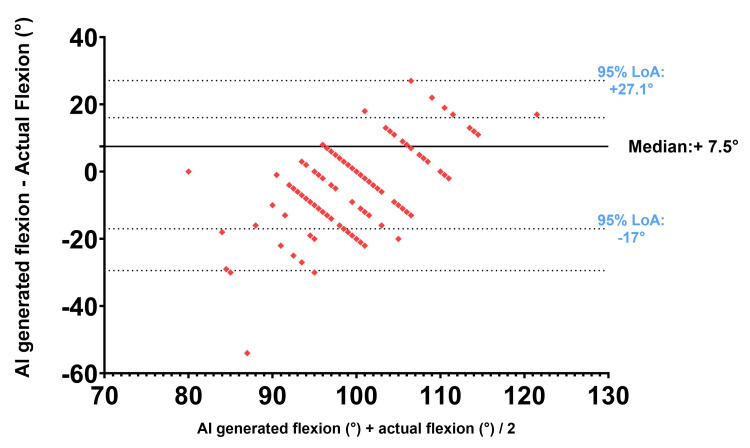
Bland-Altman plot of AI-generated knee flexion vs. actual knee flexion at six weeks post operation Difference (AI-generated knee flexion − actual knee flexion) against average (AI-generated knee flexion + actual knee flexion / 2); LoA: Limits of agreement

A non-parametric Bland-Altman analysis [9] was performed between measured knee flexion and AI-generated knee flexion. The median bias was +7.5°. The 95% limits of agreement ranged from -17.0° to +27.1°. This is shown in Figure 2. 

Difference between age ranges

**Figure 3:**
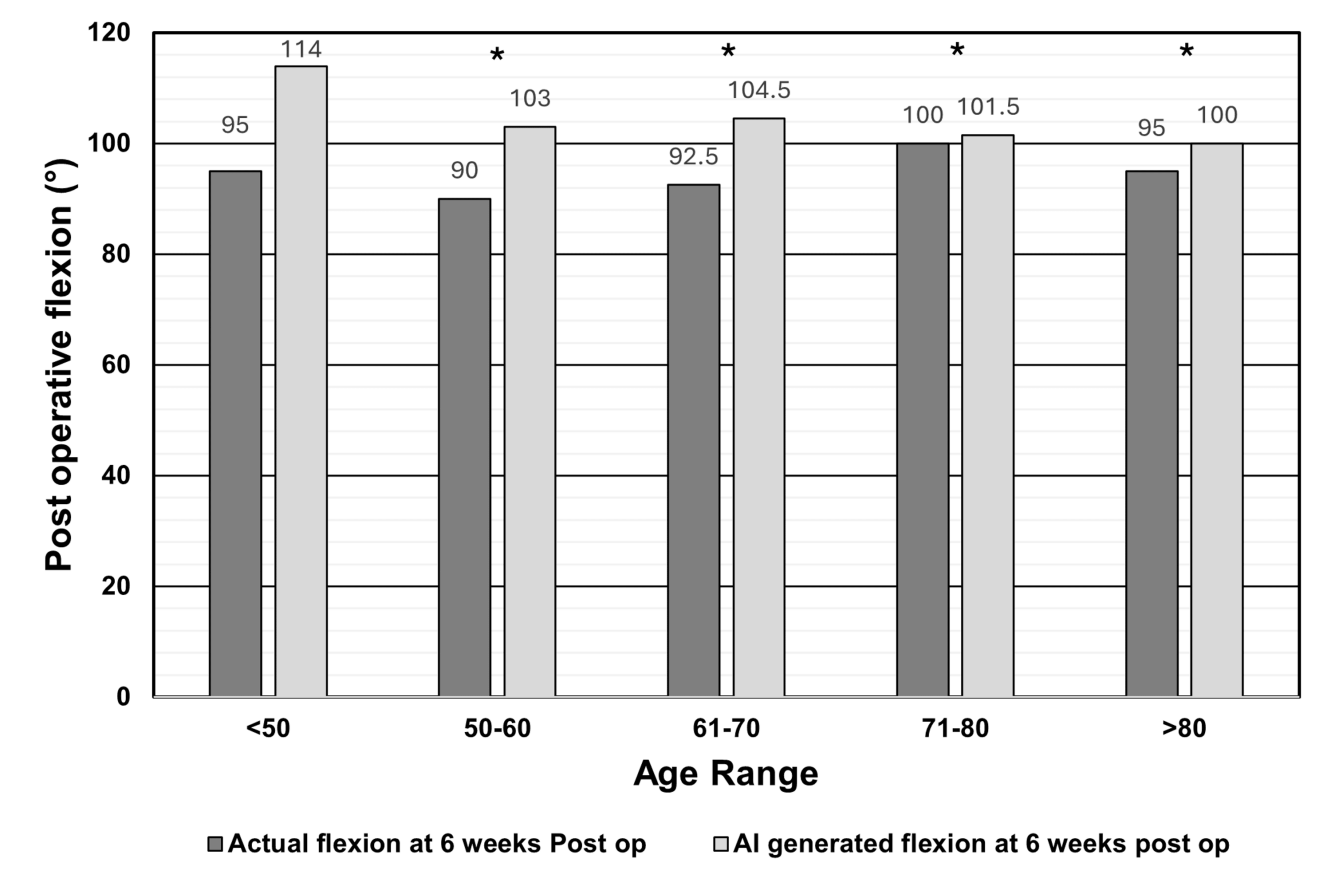
Actual six-week postoperative knee flexion vs. AI-generated six-week postoperative knee flexion by age group The bar chart compares the median actual postoperative knee flexion as recorded at the six-week postoperative clinic vs. the AI-generated six-week postoperative knee flexion grouped by age group. Age < 50 (n = 3), ages 51 to 60 (n = 19), ages 61 to 70 (n = 50), ages 71 to 80 (n = 66), age > 80 (n = 22); *p-value < 0.0001

The Wilcoxon signed-rank test was used to determine if there were significant differences between age ranges. The GPT-5 mini overestimated flexion in all age groups, with significant differences in ages 50 to 60 (actual median 90° vs. AI prediction 103°, p < 0.0001, |r| = 0.65), 61 to 70 (92.5° vs. 104.5°, p < 0.0001, |r| = 0.65), 71 to 80 (100° vs. 101.5°, p < 0.0001, |r| = 0.35), and >80 years (95° vs. 100°, p < 0.0001, |r| = 0.49). Overestimation in patients <50 years was not statistically significant (p = 0.25, |r| = 0.93). This is shown in Figure 3.

Comorbidities and ASA grade

The Wilcoxon signed-rank test showed that ChatGPT significantly overestimated flexion in non-diabetic patients by 11° (p < 0.001, |r| = 0.55), in diabetic patients by 4° (p < 0.001, |r| = 0.28), in smokers by 4° (p < 0.001, |r| = 0.30) and in non-smokers by 10° (p < 0.001, |r| = 0.61). Median absolute error significantly differed across ASA grades: 17° (I), 9° (II), and 6° (III). The Kruskal-Wallis analysis showed that absolute error significantly differed between ASA grades (p < 0.001, η² = 0.15). The BMI showed no significant association with error (r = -0.083 (95% CI -0.240 to 0.078), p = 0.296).

## Discussion

The GPT-5 mini consistently overestimated six-week postoperative knee flexion following TKR with a median bias of +7.5° and a median absolute error of 10°. The Bland-Altman analysis showed poor agreement and wide limits of variability. These findings indicate that GPT-5 mini is not precise at generating six-week postoperative knee flexion after TKR.

Prediction accuracy varied according to patient characteristics. The largest errors occurred in younger and healthier patients, whereas older or more comorbid patients (ASA III) showed smaller differences between predicted and actual flexion. Subgroup analyses further supported this pattern: ChatGPT was less accurate in predicting knee flexion for non-diabetic and non-smoking patients compared with those who were diabetic or smokers. Prediction error also varied across ASA grades, decreasing with increasing ASA grade. Interestingly, BMI showed no significant correlation with error, indicating no observable association between BMI and prediction accuracy in this cohort. The results suggest that the model performs better for patients with greater comorbidities and struggles with those who are younger and are less medically complex. This pattern mirrors existing evidence that postoperative knee flexion in TKR is influenced by age, comorbidity burden, and baseline functional reserve, with older or higher-ASA patients often achieving more predictable recovery trajectories [3-5].

These results highlight the limitations of using GPT-5 mini for generating precise numerical clinical predictions. Its lack of exposure to local postoperative range-of-motion datasets specific to TKR may have contributed to the systematic overestimation observed in this study. Although previous work has explored postoperative prediction using AI, these studies have relied on machine-learning (ML) models, rather than general-purpose LLMs such as the GPT-5 mini. For example, one study predicting walking limitation six months after TKR found that ML performed similarly to traditional logistic regression, suggesting that even specialized models may face challenges when predicting postoperative function [10]. By contrast, another study demonstrated that ML models incorporating perioperative biopsychosocial variables achieved superior predictive accuracy for patient-reported outcomes compared with logistic regression [11].

Importantly, however, no published studies to our knowledge have evaluated the use of LLMs such as the GPT-5 mini to generate numerical predictions of early postoperative knee flexion. This gap underscores the novelty and exploratory nature of the present work. The majority of TKR outcome-prediction research utilizes ML or deep-learning models trained on detailed clinical, imaging, or biomechanical datasets [10,11], whereas the GPT-5 mini draws from published literature. Therefore, any suggestion that the GPT-5 mini internalizes demographic or literature-derived “bias” remains speculative and cannot be directly tested within the current study.

Taken together, these findings indicate that the GPT-5 mini should not be used to generate numerical predictions of postoperative knee flexion or to inform clinical counselling or decision-making in patients undergoing TKR.

Limitations

This study is limited by its retrospective design and modest sample size, which may reduce generalizability and restrict subgroup power. Important variables such as preoperative flexion, prosthesis type, complications, and patient-reported outcomes were not collected. The timing of initiation of postoperative physiotherapy was not documented and therefore could not be analyzed, despite its potential influence on postoperative knee flexion. Actual knee flexion recorded in the clinic, although assessed by an experienced surgeon, did not use goniometric instrumentation and was performed without a formal reliability evaluation. Predictions were collected by a single unblinded assessor. Postoperative complications were not included as inputs into GPT-5 mini, which may have influenced predicted postoperative knee flexion. Baseline functional movement data and validated outcome scores (e.g., Oxford Knee Score) were unavailable due to institutional practice. Only one early postoperative time point was assessed, limiting evaluation of longer-term trends.

## Conclusions

The GPT-5 mini is not precise in generating six-week postoperative knee flexion after TKR. It consistently overestimated six-week postoperative knee flexion following TKR, with variability across patient subgroups. Prediction errors were greatest in younger, healthier patients and smallest among those with higher ASA grades and more comorbidities. These patterns suggest that the model may be better aligned with more medically complex patients, but its overall performance remains insufficient for clinical use.

Although ChatGPT demonstrated moderate accuracy, the magnitude and inconsistency of its errors indicate that it cannot reliably support postoperative planning or individualized rehabilitation decisions. The findings also highlight the limitations of using general-purpose language models for numerical functional prediction without training on institution-specific clinical data.

Future research should involve larger, prospective cohorts and incorporate additional variables such as preoperative function, prosthesis type, and patient-reported outcomes. Purpose-built models trained on local recovery datasets may offer greater precision and clinical relevance. Until such tools are rigorously validated, the GPT-5 mini-generated flexion estimates should not be used to guide postoperative management.
